# Food chain without giants: modelling the trophic impact of bowhead whaling on little auk populations in the Atlantic Arctic

**DOI:** 10.1098/rspb.2024.1183

**Published:** 2024-08-21

**Authors:** Amaury Thepault, Ana S. L. Rodrigues, Laetitia Drago, David Grémillet

**Affiliations:** ^1^ CEFE, Univ Montpellier, CNRS, EPHE, IRD, Montpellier, France; ^2^ Mécanismes adaptatifs et évolution (MECADEV UMR 7179), Muséum National d’Histoire Naturelle, Centre National de la Recherche Scientifique, Brunoy, France; ^3^ Laboratoire d’Océanographie de Villefranche-sur-mer, Sorbonne Université, Villefranche-sur-mer, France; ^4^ Sorbonne Université UMR 7159 CNRS-IRD-MNHN, LOCEAN-IPSL, Sorbonne Université, Paris, France; ^5^ Percy FitzPatrick Institute of African Ornithology, University of Cape Town, Cape Town, South Africa

**Keywords:** deterministic modelling, ecological baselines, historical ecology, seabirds, trophic niche, whales

## Abstract

In the Atlantic Arctic, bowhead whales (*Balaena mysticetus*) were nearly exterminated by European whalers between the seventeenth and nineteenth centuries. The collapse of the East Greenland–Svalbard–Barents Sea population, from an estimated 50 000 to a few hundred individuals, drastically reduced predation on mesozooplankton. Here, we tested the hypothesis that this event strongly favoured the demography of the little auk (*Alle alle*), a zooplanktivorous feeder competitor of bowhead whales and the most abundant seabird in the Arctic. To estimate the effect of bowhead whaling on little auk abundance, we modelled the trophic niche overlap between the two species using deterministic simulations of mesozooplankton spatial distribution. We estimated that bowhead whaling could have led to a 70% increase in northeast Atlantic Arctic little auk populations, from 2.8 to 4.8 million breeding pairs. While corresponding to a major population increase, this is far less than predicted by previous studies. Our study illustrates how a trophic shift can result from the near extirpation of a marine megafauna species, and the methodological framework we developed opens up new opportunities for marine trophic modelling.

## 1. Introduction

The earliest and most prominent impact of the Anthropocene is the consistent removal of megafauna from ecosystems colonized and exploited by human societies [1–3]. It is now well established that megafauna plays a critical role in biosphere functioning [4], supporting a wide range of ecological processes, such as landscape structuring [5], seed dispersal [6], nutrient recycling [7] and regulation of trophic networks [8]. Understanding the ecological consequences of past megafaunal collapses is therefore essential to the conservation and management of today’s ecosystems. Indeed, our current understanding of biosphere functioning mostly stems from the observation of ecosystems that have been depleted of their megafauna for decades, centuries or even millennia, and which, due to resulting trophic cascades, are altered states resulting from past anthropogenic regime shifts [9,10]. In this context, estimating historical baselines enables us to improve conservation strategies and better predict, inform and anticipate the ecological consequences of the demographic recovery of formerly exploited populations [9]. Failure to do so could lead to inaccurate interpretations, resulting from the well-known ‘shifting baseline syndrome’ [11], such as attributing demographic changes to recent threats rather than to a rebound of the ecosystem towards a previous state.

In marine ecosystems, where human presence has long been constrained by technological capabilities, the removal of megafauna is more recent than in terrestrial ecosystems [12]. The collapse of whale populations is a relevant example: although whaling dates back to antiquity, it intensified in western Europe in the eleventh century and expanded worldwide from the seventeenth century onwards [13]. Initially targeting slow, coastal whales with high buoyancy, whaling gradually broadened its scope to include a wide range of species, a transition made possible by technological innovations in the context of increased international competition [13]. Despite the 1985 global moratorium on whaling, nearly 30% of cetacean species assessed by the International Union for Conservation of Nature (IUCN) are still listed as threatened [14], with the abundance of large whales estimated at only 36% of their historical value [15].

Although the whaling industry did not result in the global extinction of any whale species, the functional extinction of many populations likely had profound ecological consequences. Indeed, whales play key roles in marine ecological processes through vertical mixing, horizontal transfer, recycling of nutrients and with whale carcasses conveying organic matter to the seafloor [16]. For example, in the Southern Ocean, pre-whaling primary production may have been 20% higher than today because of nutrient recycling, in particular iron fertilization, promoted by the much larger historical whale populations [17]. The top-down whale control of Southern Ocean krill (*Euphausia superba*) populations through predation and fertilization led Smetacek [18] to develop the evocative concept of a ‘food chain of giants’ [18].

Competition release triggered by the functional extinction of whales in several areas of the world vacated trophic niches that could have benefited competing species, redirecting a significant proportion of energy flows [19]. However, the extent of this historical phenomenon depended on the ability of competitors to fill the whales’ trophic niches. This shift is all the more uncertain as whales are characterized by an astonishing and often underestimated capacity to prospect and filter their environment [17]. They also have a metabolic efficiency specific to large organisms, resulting from an evolutionary trajectory that ensures the energetic profitability of foraging under conditions of unequal distribution of their resources in space and time [20,21]. The complex interplay between predation release and competition release is currently a major unknown in the trophic consequences of large whale population collapses. To shed light on this interplay, it is necessary to assess the degree of overlap between the trophic niches of whales and competing species.

In the Southern Ocean, the conversion of predation release into competition release forms the bedrock of the ‘krill surplus hypothesis’, which postulates that whaling has led to an increase in the populations of seals, seabirds and fish. On the other hand, the ‘krill paradox hypothesis’ suggests that krill productivity has dwindled over the last few centuries, mainly due to a decline in iron recycling through whale faeces [17], leading to a stagnation or decline in the abundance of these competing species. Yet, ecosystem mass-balance models (e.g. Ecopath), used to test these two hypotheses, assume full overlap between the trophic niches of these predators. While overlap is confirmed with respect to their diets [22], it appears to be limited by differences in their respective spatial, vertical and temporal use of the environment [23–25], and by disparate abilities to exploit prey fields [22].

On the other side of the planet, in the Atlantic Arctic, zooplankton resources are jointly exploited by one of the largest and now rarest marine mammals on Earth, the bowhead whale (*Balaena mysticetus*), and by one of the smallest and most abundant seabirds, the little auk (*Alle alle*), the latter being 500 000 times lighter than the former. Assessing trophic niche overlap between these two species is essential to understand the interplay between predation release and competition release that shaped the consequences of whaling on Artic ecosystem functioning. Although both species share a similar diet, they exhibit radically contrasting foraging behaviours regarding filtration volume, foraging depth, prey selectivity and capture technique.

Bowhead whales exhibit year-round foraging with peaks in the summer and autumn [26,27], and an exclusively zooplanktivorous diet [28]. They are continuous filter feeders, swimming at low speed with their mouths open, in contrast to the engulfment-based foraging strategy of rorquals [29]. Bowhead whale foraging is essentially non-selective and is thought to be based on the filtering of more than 80 000 m^3^ of water per day. To meet its energy needs, the bowhead whale prospects its environment, from the surface to depths rarely exceeding 200 m [30], feeding on patches of zooplankton. In the North Atlantic, the diet of bowhead whales consists predominantly of mesozooplankton—copepods of the genus *Calanus*, in particular *C. finmarchicus*, *C. hyperboreus* and *C. glacialis* [29,31].

The East Greenland–Svalbard–Barents Sea bowhead whale population (EGSB) is the first to have been intensively whaled, and the one for which whaling led to the most dramatic and the most long-lasting depletion [32]. Before industrial whaling started in 1610, the EGSB population extended from northern Iceland to Novaya Zemlya [33], with an estimated population of about 50 000 individuals [34]. Three hundred years of intensive persecution [34] resulted in its commercial extinction [35], with perhaps just a few dozen individuals remaining by 1910 [36]. Despite no whaling throughout most of the twentieth century, the EGSB population remains extremely rare [36]. With less than 250 mature individuals, it is listed as endangered by the IUCN [37] and is only now showing the very first signs of recovery [32]. This long-term population depletion likely released the predation pressure on mesozooplankton. Yet, in great contrast with the Southern Ocean, Arctic primary productivity is mainly limited by nitrogen availability [38] and not by iron, ruling out the possibility that mesozooplankton productivity declined due to a cessation of the whale nutrient pump. In this region, the ‘fish and seabird success scenario’ is more plausible [39], with the collapse of the whale population likely leading to an increase in the abundance of direct competing species, primarily the little auk (*A. alle*), polar cod (*Boreogadus saida*) and capelin (*Mallotus villosus*), as a result of competition release.

The little auk, a member of the Alcidae family, is currently the most abundant seabird species in the Atlantic Arctic, with an estimated overall population of between 40 and 80 million individuals [40], and about 4.8 million breeding pairs across the historical range of the EGSB population of bowhead whales [41]. This small diving bird, weighing on average 160 g, has an exclusively zooplanktivorous diet, similar to the diet of bowhead whales [42,43]. Due to their abundance, trophic position and active transfer of organic matter to coastal ecosystems, little auks play a major ecological role in Arctic ecosystems [44], being considered as ecosystem engineers [45]. They forage underwater along wing-propelled dives mainly to depths <20 m [42,43] and undertake visually guided suction feeding through the extension of their gular pouch [46].

Here, we tested the plausibility of the ‘seabird success scenario’ following bowhead whaling in the northeast Atlantic Arctic. We predicted that the competition release resulting from whale hunting could have resulted in a significant demographic growth of little auks. To test this hypothesis and estimate the abundance of little auks in an Arctic food chain with giants (i.e. with pre-whaling bowhead numbers), we modelled the trophic niches of little auks and bowhead whales using deterministic simulations of mesozooplankton distribution in the study area. This allowed us to assess the trophic niche overlap between the two species and to estimate the quantity of released mesozooplankton biomass that little auks may have benefited from with whaling.

## 2. Methods

### (a) Study area

**Figure 1.**
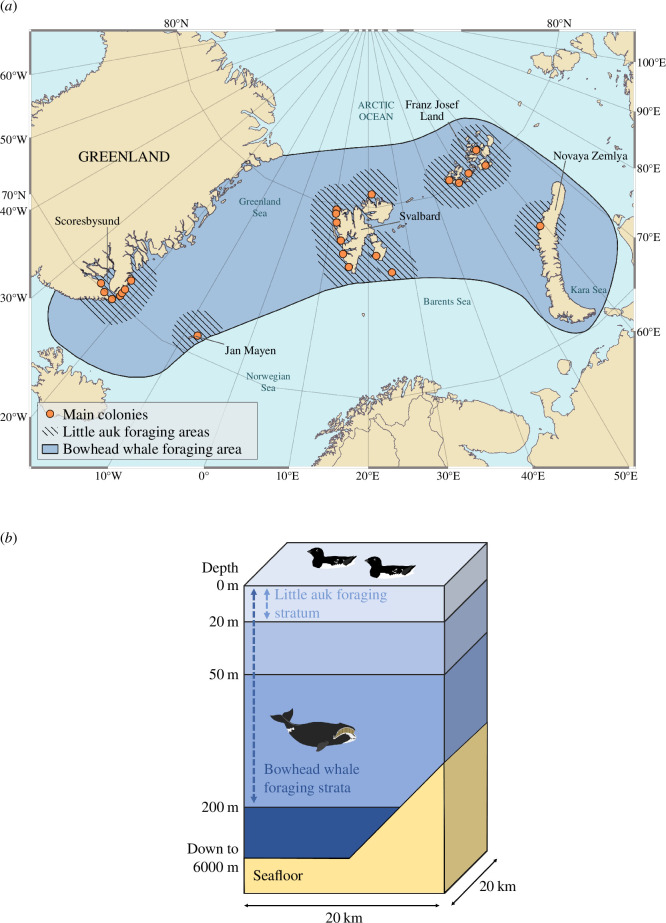
Horizontal and vertical structure of the study area. (*a*) Map of the study area with the summer foraging areas of bowhead whales and little auks. (*b*) Vertical cell structure, with three strata and the foraging depth of each predator species.

The study area covers 2 317 000 km², including the Greenland Sea, the northern Barents Sea, the southwestern Kara Sea and part of the Arctic Ocean basin (figure 1*a*), and corresponds to the pre-whaling summer foraging range of the EGSB bowhead whale population [33]. Within this study area, we plotted circles with a 150 km radius around the main little auk colonies [41] to estimate a 559 000 km² foraging area for this species during its breeding season. This radius is based on studies of sightings of individuals at sea and GPS-tracking data of breeding adults [41,42]. We structured the study area into three dimensions (latitude, longitude and depth) using cells with a surface area of 400 km² (20 × 20 km), divided vertically into three depth strata: 0−20 m, 20−50 m and 50−200 m (figure 1*b*). Each combination of a cell and a stratum forms a parallelepiped hereinafter called a ‘water mass’ and denoted m .

### (b) Input data

This study used as input: spatially explicit estimates of copepod biomass across the study area [47]; a number of parameters extracted from the literature that characterize mesozooplankton production, as well as the abundance and feeding ecology of little auks and of bowhead whales (listed in electronic supplementary material, table S1).

### (c) Model construction and simulations

To estimate the effect of bowhead whaling on the abundance of little auks, we developed a *sui generis* model consisting of three modules (electronic supplementary material, figure S1). The first module is a deterministic model of mesozooplankton distribution in the study area. The second module is a trophic model that analyses niche overlap based on the foraging capacities of bowhead whales and little auks under previously simulated conditions of mesozooplankton distribution. The third module consists of a non-dynamic food web model backcasting the demographic increase of little auks resulting from whaling by transposing currently observed competitive conditions into a theoretical Arctic food web which differs from today’s only in having the bowhead whale population at pre-whaling levels. For simplicity, this food web is hereafter referred to as ‘seventeenth century’, although it assumes that the only ecosystem-level change from the seventeenth to the twenty-first centuries was in bowhead whale abundance and ignores the potential impacts of climate change or fisheries.

In this model, we assumed that the current population of little auks $N_{a_{21\;}}$ comprises 4.8 million breeding pairs in the study area (standard deviation (s.d.): 300 000) [41] and that the EGSB population of bowhead whales was 50 000 individuals (s.d.: 2500) in the seventeenth century ( $N_{b_{17}}$ ) [34] and 343 individuals (s.d.: 50) in the twenty-first century ( $N_{b_{21}}$ ) [32,48].

We simulated mesozooplankton predation during the little auk breeding season, from mid-May to mid-August (duration n = 92 days) [42], a period characterized by the simultaneous presence of both species in the study area. We made this choice with the dual aim of simplifying the system by ignoring the spatial dynamics of bowhead whale and little auk migration, and targeting the season with the highest foraging demand from these two species. Indeed, bowhead whales have an annual foraging cycle, with peak consumption in the summer and autumn required to store lipid reserves [26,27,49], whereas little auks have particularly high energy requirements during the summer due to chick feeding and associated travel costs [43,50].

This model further simplifies the dynamics of the study ecosystem in one important way. It assumes that the interspecific relationship between bowhead whales and little auks takes place solely via the consumption of mesozooplankton at the overlap of their trophic niches, without considering potential behavioural interactions between the two species (e.g. facilitation in food searching or spatial avoidance).

#### (i) Modelling the distribution of mesozooplankton biomass (Module 1)

We modelled the distribution of mesozooplankton biomass in three steps (electronic supplementary material, methods S1 and figure S2). First, we modelled the horizontal spatial distribution (latitude and longitude) of the biomass produced during the study season in the water column of each cell. To do this, we used a recently published global estimation of copepod biomass generated by habitat modelling [47]. In this study, the authors employed supervised machine learning algorithms based on boosted regression trees to estimate the biomass distribution of several zooplankton taxa including copepods, using quantitative *in situ* imaging data and environmental climatologies (electronic supplementary material, methods S1). In our study, we converted these copepod biomass estimates to production estimates of mesozooplankton biomass over the study period (electronic supplementary material, equation S2). Secondary production of mesozooplankton is known to exhibit highly discontinuous temporal dynamics, characterized by extended periods of biomass stagnation and short periods of high production [51,52]. In the absence of knowledge on foraging strategies in relation to secondary production, we assumed that predators (e.g. bowhead whale, little auk, polar cod, capelin) target mesozooplankton patches when a period of high productivity has just occurred. Hence, instead of modelling average biomass as estimated by [47], our aim was to model biomass peaks. This is the reason why we chose to model biomass production across the study period, rather than mean biomass at a given time. This assumes that the mesozooplankton biomass of each cell is exposed to a single act of foraging during the study period.

Second, we modelled the vertical distribution of the biomass in the water column of each cell, by calculating its distribution in each of the three strata (0−20, 20−50 and 50−200 m) based on data from mesozooplankton stratification studies off the coasts of Svalbard [53,54]. This results in an estimate of biomass per water mass m (electronic supplementary material, methods S1).

Third, we modelled the heterogeneity of mesozooplankton density within each water mass m , assuming that mesozooplankton is distributed into two types of structures: (i) mesozooplankton patches, i.e. volumes of water with a high density of individuals resulting from the discontinuous distribution of organisms in the marine environment, due to a combination of physical and biological factors [55], and (ii) background, i.e. volumes of water defined in contrast to patches and with a diffuse density of individuals. Previous studies have shown that bowhead whales and little auks target high-density mesozooplankton patches to meet their energy needs [43,56]. We used a deterministic and non-dynamic approach to model mesozooplankton patches. Our approach is deterministic because we modelled patches using two patchiness intensity parameters estimated from *in situ* observations made using optical particle counter (OPC) technologies off Svalbard [57] (electronic supplementary material, methods S2). Furthermore, our approach is non-dynamic because we assumed that patch characteristics (location, depth and density) are constant in time and space from mid-May to mid-August. This assumption excludes the effects of advection [58], seasonal vertical migration [59] and dispersal [60] on the distribution of mesozooplankton during the study period.

#### (ii) Modelling trophic niche overlap (Module 2)

This second module assesses the overlap between the trophic niches of bowhead whales and little auks in the study area from mid-May to mid-August (electronic supplementary material, methods S3). We define trophic niche overlap as the fraction of an *n*-dimensional hypervolume in which several species coexist by exploiting similar resources.

We first estimated the total biomass of mesozooplankton accessible to a predator x ( $Q_{a}$ for little auks and $Q_{b}$ for bowhead whales), by projecting its trophic niche into the simulated conditions of mesozooplankton distribution as per Module 1. Unlike mass balance models, which generally assume total accessibility of prey by predators in a given trophic system [61], biomass accessibility is here conditioned by three criteria, which together define each species’ trophic niche: (i) the latitude and longitude of the cell relative to the predator’s foraging area (figure 1*a*), (ii) the depth of the stratum relative to the predator’s foraging depth (figure 1*b*) and (iii) the density of mesozooplankton biomass in the patches relative to a foraging density threshold specific to each species. We estimated this foraging density threshold to be 9.4 g m^−3^ (s.d.: 1) for bowhead whales and 13.7 g m^−3^ (95% prediction interval: 7.8–22.2) for little auks (electronic supplementary material, methods S4 and figure S3).

By comparing the biomass accessible to each predator within each water mass and adding the common biomass across water masses, we calculated the amount $Q_{s}$ of mesozooplankton biomass common to both trophic niches. From this, we then derived: $W=Q_{s}/Q_{a}$ , the degree of overlap (%) of the little auk trophic niche by bowhead whales and $Y=Q_{s}/Q_{b}$ , the degree of overlap (%) of the bowhead whale trophic niche by little auks. In addition, we calculated the consumption $C_{b_{17}}$ and $C_{b_{21}}$ of biomass by bowhead whales in the seventeenth and twenty-first centuries and the consumption $C_{a_{21}}$ of biomass by little auks in the twenty-first century (electronic supplementary material, methods S5).

#### (iii) Simulating little auk abundance with pre-whaling bowhead numbers (Module 3)

In this third module, we deemed the current abundance of little auks to be the result of their ability to compete within their trophic niche during the breeding season with other zooplanktivorous species (e.g. Arctic cod, capelin). This is based on the assumption that the summer availability of mesozooplankton biomass is the main factor limiting the demography of little auks in the northeast Atlantic Arctic, due to their tight energy balance, heightened during the breeding season with the feeding of the chick [43].

We estimated the little auk abundance with pre-whaling bowhead numbers ( $N_{a_{17}}$ ) in three steps. First, we calculated the parameter $Z_{a}$ (%), as the fraction of biomass consumed by little auks in relation to the total that was accessible to them and not consumed by bowhead whales (equation (2.1)).


(2.1) $$Z_{a}=\;\frac{C_{a_{21}}}{Q_{a}-\;\left(C_{b_{21}}\times \;Y\right)}$$

We considered this proportion $Z_{a}$ to be an indicator of the ability of little auks to exploit their trophic niche in a multi-species competitive environment. This parameter allows us to take into account competition with other zooplanktivorous species other than bowhead whales, even though we did not explicitly model them. We estimated this parameter based on twenty-first century conditions and then assumed it applied equally to the seventeenth century, i.e. that the competitive conditions did not change between the two time periods.

Second, we estimated the quantity of ‘released’ mesozooplankton biomass due to whaling, as the difference between the total consumption by bowhead whales in the seventeenth and the twenty-first centuries ( $C_{b_{17}}-\;C_{b_{21}}$ ). We considered this ‘released’ biomass to be accessible to current populations of little auks only with a ratio equivalent to the degree of overlap Y of the bowhead whale trophic niche by little auks.

Finally, we estimated the little auk abundance with pre-whaling bowhead numbers (via cross-multiplication) by applying simulated twenty-first century competitive conditions to the seventeenth century food web, i.e. by considering that little auks, facing competition from other predators, consume only a share $Z_{a}$ of this additional accessible biomass (equation (2.2)).


(2.2) $$N_{a17}=\;N_{a21}\times \;\frac{C_{a_{21}}-\;\;\left[\left(C_{b_{17}}-\;C_{b_{21}}\right)\;\times \;Y\;\times \;Z_{a}\right]}{C_{a_{21}}}$$

#### (iv) Simulations and analysis of variance

We built our model upon a set of 23 input parameters related to the distribution and ecology of zooplankton, little auks and bowhead whales, whose values we extracted or estimated from the literature (electronic supplementary material, table S1). To quantify the uncertainty around our estimates of ${N_{a}}_{17}$ , we conducted Monte Carlo simulations, by running 10 000 independent iterations of the calculations, each with a combination of input parameter values determined by random selection from the associated distribution laws. For 19 parameters, we allowed them to vary within normal distributions defined by a mean and s.d. We also allowed the two patchiness intensity factors to vary within distribution laws in each water mass m, in order to reflect environmental heterogeneity. The two remaining parameters were set to fixed values. In addition, we allowed the copepod biomass estimates to vary based on the mean and s.d. calculated at the spatial resolution of 1° of latitude and longitude as provided by [47]. We considered as unrealistic any iterations that failed due to an insufficient biomass accessible to at least one of the predator species and discarded them from the analyses (electronic supplementary material, methods S3).

Following White *et al*. [62], we quantified the proportion of variance in our final estimate of little auk abundance in an Arctic food chain with pre-whaling bowhead numbers $N_{a_{17}}$ explained by each parameter of the model using an ANOVA constructed with all parameters.

## 3. Results

According to our calculations, the mesozooplankton biomass in the study area had an average density of 33.2 g m^−2^ (95% prediction interval: 28.5–38.9) and 0.14 g m^−3^ (0.13–0.16). From mid-May to mid-August, an estimated *B* = 126.3 megatonnes (Mt) (94.7–168) of mesozooplankton were produced in the bowhead whale foraging area between 0 and 200 m depth. Mesozooplankton patches accounted for 8.4% (8.3–8.5%) of the water volume in the study area. These patches concentrated 52.4 Mt (39.3–69.7) of mesozooplankton biomass produced during the study period, i.e. an average of 41.5% of the total production, with a post-production mean density of 5.4 g m^−3^ (4.0–7.3) in the 0−20 m stratum, 3.1 g m^−3^ (2.3–4.2) in the 20−50 m stratum and 0.5 g m^−3^ (0.3–0.7) in the 50−200 m stratum.

**Figure 2.**
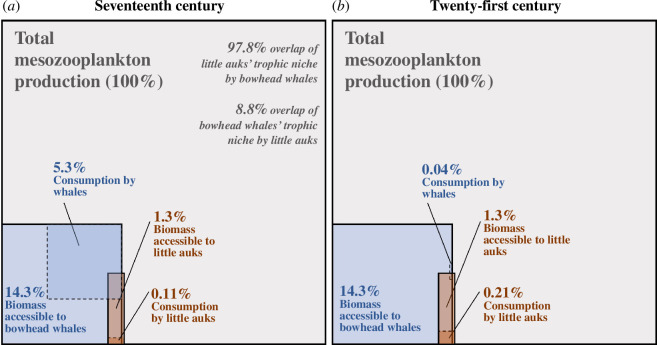
Simulated food consumption of bowhead whales and little auks within simulated mesozooplankton production, in (*a*) the seventeenth century (with pre-whaling bowhead numbers) and (*b*) the twenty-first century. In both panels, the larger squares represent total mesozooplankton production from mid-May to mid-August within the study area between 0 and 200 m depth. Smaller polygons represent the average fraction of biomass accessible to each species (blue for bowhead whales; orange for little auks) and then within those the fraction consumed (in darker colours). Tropic niche overlap between species is represented by overlap between polygons.

We estimated that $Q_{b}$ = 18.7 Mt (7.4–39) of mesozooplankton is accessible to bowhead whales, which is on average 14.3% of the secondary biomass production (*B*) from mid-May to mid-August in the study area (figure 2). In the seventeenth century, bowhead whales consumed $C_{b_{17}}$ = 6.5 Mt (4.3–8.9) of mesozooplankton (5.3% of *B*). In the twenty-first century, consumption by bowhead whales fell to $C_{b_{21}}$ = 0.04 Mt (0.03–0.07) (0.04% of *B*). Of the biomass that is accessible to them, bowhead whales thus consumed on average 42% (15.1–89%) in the seventeenth century and 0.3% (0.1–0.6%) in the twenty-first century. By comparing consumption between the seventeenth and twenty-first centuries, we calculated that whaling led to a predation release of 6.46 Mt (4.26–8.89) of mesozooplankton.

Concerning little auks, we estimated that they have access to $Q_{a}$ = 1.69 Mt (0.32–4.82) of mesozooplankton (1.3% of *B*; figure 2), of which they consume $C_{a_{21}}$ = 0.26 Mt (0.22–0.31) (0.21% of *B*), which represents a share $Z_{a}$ = 25.6% (5.3–81%) of the biomass they have access to after consumption by bowhead whales.

Our results indicate that the trophic niches of the two species overlap by an average of $Q_{s}$ = 1.61 Mt (0.32–4.34) of mesozooplankton biomass, which corresponds to W *=* 97.8% (70.4–100%) of the little auks trophic niche and Y = 8.8% (2.2–17.0%) of the bowhead whales trophic niche. Eighty-three per cent of the discrepancy between the two trophic niches is explained by geographical differences in foraging areas, 9% by differences in foraging depth and 8% by differences in the threshold density of mesozooplankton biomass.

Of the biomass ‘released’ by whaling, only 0.55 Mt (0.13–1.22) is within the trophic niche of little auks. By applying simulated twenty-first century competitive conditions to a simplified seventeenth century food web, we calculated that an additional 0.11 Mt (0.04–0.23) of mesozooplankton was consumed by little auks after whaling, compared with the seventeenth century.

**Figure 3.**
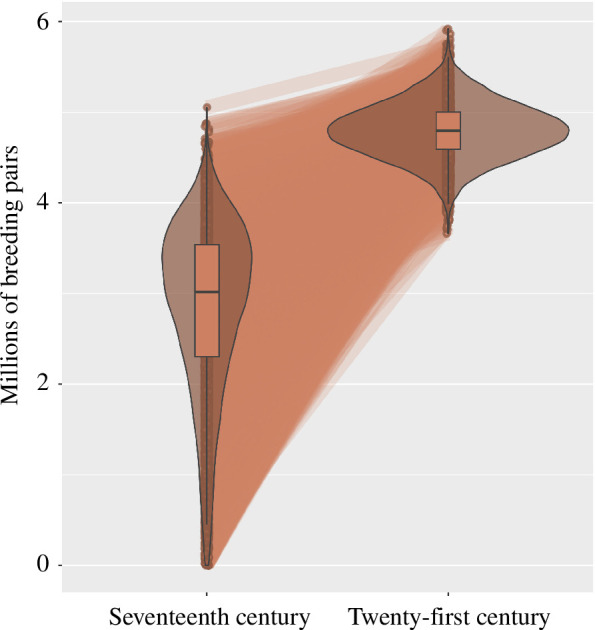
Simulations of little auk abundance within the study area in the seventeenth century (with pre-whaling bowhead whale numbers) and in the twenty-first century. The shape connecting the two violin distributions represents the cumulative sum of 10 000 simulated little auk demographic trajectories in response to bowhead whaling.

Using equation (2.2), we estimated that, in an Artic food chain with pre-whaling bowhead whale numbers, little auk abundance in the northeast Atlantic Artic could be as low as $N_{a_{17}}$ = 2.8 million breeding pairs (0.6–4.2), compared with a mean of $N_{a_{21}}$ = 4.8 million breeding pairs at the beginning of the twenty-first century (figure 3). We thus estimate that the decline in bowhead whale numbers due to whaling could have resulted on average in a 70% increase in little auk abundance.

Our model’s results indicate that most of the biomass accessible to bowhead whales was located in the Greenland Sea (64.5% (60.1–67.9%)), followed by the Barents Sea (17.8% (14.7–21.2%)), the Arctic Basin (7.8% (6.7–8.9%)) and the Kara Sea (7.5% (6.4–8.8%)) (electronic supplementary material, figure S4), with a vertical distribution strongly structured towards the surface, with 12.8 Mt (5.4–24.2) in the 0–20 m stratum, 5.3 Mt (1.4–14.1) in the 20–50 m stratum and 0.6 Mt (0.1–1.6) in the 50–200 m stratum (electronic supplementary material, figure S5). For little auks, accessible biomass was mainly located off Svalbard (42.2% (35.9–50.5%)) and the Scoresby Sund in eastern Greenland (20.7% (14.3–26.3%)), around Jan Mayen (16.4% (11.1–23.2%)), Franz Josef Land (16.1% (9.2–22.2%)) and Novaya Zemlya (4.7% (2.5–9.2%)) (electronic supplementary material, figure S4).

Among the 10 000 Monte Carlo simulations, we discarded 12.4% from the analyses for producing unrealistic scenarios. According to our sensitivity analysis, 54.4% of the variance in our estimate of little auk abundance with pre-whaling bowhead whale numbers is explained by two parameters regulating mesozooplankton production: the production to biomass ratio (32% of the variance) and the proportion of annual secondary production achieved during the study period (22.4% of the variance) (electronic supplementary material, figure S6).

## 4. Discussion

### (a) Ecological implications

Our findings indicate that competition with bowhead whales had a substantial effect on little auk populations: we estimate that the near extinction of the former might have led to a 70% increase of the latter in the northeast Atlantic Arctic. Our study, therefore, supports the prevailing hypothesis that the current high abundance of little auks is, at least partly, the result of historical whaling. However, we estimate a much less impressive demographic growth than previously suggested in the scientific literature [39,63]. The single previous study that attempted to quantify the impact of bowhead whaling on Arctic marine food webs estimated that the Svalbard little auk population increased by a factor of 2–10 [39].

Our lower estimate results mainly from the modelling of a broader trophic niche of bowhead whales than in that previous study, as we found they have access to mesozooplankton resources across the entire study area (and not just near Svalbard as in [39]) and thus probably forage far beyond the reach of little auks (electronic supplementary material, figure S4). The modelling of this broader trophic niche leads to two subsequent results. First, given a historical population of 50 000 individuals, we found that bowhead whales consumed 42% of the biomass accessible to them. This is far below the optimal efficiency previously suggested by modelling studies, for summer [39] or winter feeding [64]. Second, we estimated that the trophic niche of little auks covers only one-tenth of that of bowhead whales. As a consequence of these two points, the amount of ‘released’ biomass from which little auks benefited was logically constrained, resulting in a rather small biomass gain. Altogether, little auks benefited from a lower competition release compared with a scenario in which whales would exhibit optimal predation efficiency within a narrower trophic niche closer to shore, as assumed in Weslawski *et al*. [39].

The broader trophic niche of bowhead whales compared with that of little auks is almost entirely explained (92%) by differences in their respective geographic foraging area and their ability to forage at depth. It is, therefore, highly likely that other zooplanktivorous species present throughout the study area, and capable of foraging at greater depths than little auks, benefited from larger quantities of ‘released’ biomass. With regard to the ‘fish and birds success scenario’ triggered by whaling [39], the present study mitigates the extent of the ‘bird success’ component and invites us to reassess the potential success of other mesozooplankton predators.

In addition to a quantitative shift in the predation pressure of mesozooplankton, the near extirpation of bowhead whales likely resulted in important qualitative changes. With whaling, the Arctic ecosystem virtually lost a formidable zooplanktivorous predator—the bowhead whale—characterized by a non-selective foraging behaviour [29], high pelagic mobility [65] and a relatively low critical foraging threshold for prey biomass density (9.4 g m^−3^). This partly benefited another zooplanktivorous predator—the little auk—characterized by a strong coastal dependence during the breeding season, a visually guided selective foraging behaviour [42,46] and a relatively high critical foraging threshold for prey biomass density (13.7 g m^−3^). Consequently, at least in the coastal areas surrounding little auk colonies, bowhead whaling led to a qualitative change in predation pressure, from a uniform and non-selective predation pressure to a predation pressure that depends on location, copepod species, individual size and life stage [19]. While the present study modelled the whole mesozooplankton compartment as a single species, future studies could investigate the consequences of these qualitative changes in predation pressure.

Our results strongly suggest that the populations of little auks are regulated by additional factors beyond competition with bowhead whales. Indeed, in the current environment almost devoid of bowhead whales, we estimate that populations of little auks consume about one-fourth of the mesozooplankton biomass accessible to them. In our model, we assume that competition with other mesozooplankton predators (fish, macrozooplankton) [39,64] plays a substantial role in regulating their population. In addition, inter-seasonal and inter-annual variability in mesozooplankton productivity [66] may limit little auk population growth, as could nesting site availability. These potential additional factors do not affect our results, as we assume they are stable between the seventeenth and the twenty-first centuries. The true value of increase in little auk population may be different if those additional factors were in fact not stable. For example, if competition with fish predators has declined (e.g. because of the recent impact of fisheries), then the actual increase in the little auk population may have been stronger than our prediction. Conversely, if nesting site availability has decreased (e.g. because of increased competition with other seabirds), then our prediction may be an overestimate. The estimated 70% increase should thus be seen as a prediction of the effect of bowhead whale decline on the population of little auks (given the model’s assumptions), rather than an estimate of the actual change in abundance. In reality, the historical change in the population of little auks was likely different and not all attributable to whaling.

Finally, our study is relevant in the context of the ongoing recovery of the EGSB population of bowhead whales [67]. Despite the fact that its exploitation has ceased over 100 years ago, this population is recovering much more slowly than those in the Pacific Arctic and in western Greenland [67]. Our results indicate that competition with little auks is highly unlikely to pose a limit to this recovery, given the very small fraction of the whale’s niche overlapped by these seabirds (8.8%; figure 2). At current rates, it will take a very long time for bowhead whales to recover to their historical levels, but if that happens, our results predict that (everything else being equal) the populations of little auks would decline back to their pre-whaling abundance, as a consequence of stronger competitive pressure within their tropic niche. A reversal to historical conditions is, however, unlikely, as ongoing climate change is likely to affect Arctic plankton communities, with cascading effects on marine megafauna populations [68].

### (b) Future directions

The deterministic approach we used to simulate the distribution of mesozooplankton density in the water column opens up new opportunities for marine trophic modelling. This approach, made possible by the development of OPC technologies, is an alternative to the mechanistic modelling of the physical and biological factors at the origin of patchiness [60,69]. Indeed, the complexity in modelling these phenomena makes it difficult to take into account the heterogeneity in zooplankton distribution when reconstructing food webs. More frequent integration of zooplankton patchiness into trophic models, as performed in this study, would allow better consideration of the role of prey biomass critical density thresholds in the modelling of competitive interactions.

In this study, the deterministic model was based on a non-dynamic approach of the zooplankton system in time and space. In other words, we excluded the effects of advection [58], seasonal vertical migration [59] or plasticity of aggregation structures on the spatial and temporal distribution of mesozooplankton, and thus on biomass accessibility to predators. We reduced the bias associated with the first two processes by modelling the food web over a time interval (about 3 months) that is short when compared with the annual spatial dynamics and life cycle of *Calanus* species. Regarding the third process, Arctic copepods have much lower mobility than, for example, Antarctic euphausiids. They use this mobility mainly at very fine scales to escape predators, move vertically in the water column, find prey and reproduce [70,71]. Therefore, we assumed that large-scale copepod patchiness is due to extrinsic physical and biological factors (e.g. currents, areas of high primary productivity), rather than to active horizontal movement [72]. In this context, we assumed that Arctic mesozooplankton distribution is constant in time and space over a 3 month period. This non-dynamic assumption likely underestimates the amount of biomass accessible to both predators and thus the little auk abundance with pre-whaling bowhead whale numbers, hence resulting in an overestimate of the population increase.

Due to the non-dynamic nature of the model, we built it using the secondary production of mesozooplankton (from which we simulated the total biomass that predators have access to during the seabird breeding season), rather than the biomass present in the system at a given time. The latter option would have required dynamic modelling of production and predation over time. From a methodological perspective, our choice implies the integration of two parameters (the production-to-biomass ratio and the proportion of annual secondary production achieved during the study period) whose uncertainty explains more than 50% of the total variance of the model (electronic supplementary material, figure S6). From a biological perspective, this approach is based on the assumption, increasingly confirmed by *in situ* studies, of a bell-shaped dynamic in the secondary production of the Arctic zooplankton system during the summer period, consisting of a multitude of species-specific and life-stage-specific production peaks [51,52]. This strong seasonality in production has been interpreted by previous authors as the result of an evolutionary trajectory of the zooplankton system, aimed at reducing interspecific and intergenerational competition [52]. The growth of mesozooplankton patches would thus be the result of punctuated episodes of production, differentiated by species and life stage, that rapidly reach biomass densities high enough to provide a profitable resource for bowhead whales and little auks. We, therefore, assumed that secondary production is locally equivalent to biomass just after a production peak, i.e. when predation occurs. Our focus on secondary production operates under the assumption that the biomass at a given location undergoes a single act of foraging during the study period. In contrast to a dynamic model, this assumption neglects diffuse forms of predation by zooplanktivorous species (e.g. predation by macrozooplankton). As a result, it likely overestimates the biomass accessible to species with highly localized predation (including bowhead whales and little auks), resulting in an underestimate of population increase.

Our modelling exercise opens up multiple opportunities for improvement. First, whereas we assumed that little auks and bowhead whales only influence each other through interspecific competition mediated by their differential access to mesozooplankton, other forms of ecological interaction can exist between these two species, such as facilitation in food searching or spatial avoidance. These can potentially be investigated through behavioural studies in west Greenland, where little auks coexist today with higher densities of bowhead whales.

Second, we modelled zooplankton patchiness at the mesoscale (>2 km), thereby excluding the effects of microscale structures (<2 km) on the foraging behaviour of little auks and bowhead whales. Yet, given the differences in size and foraging behaviour between both species, it is conceivable that little auks target mesozooplankton patches at finer scales [73], compared with those targeted by bowhead whales, adding another factor for trophic niche differentiation potentially reducing trophic overlap.

Third, future studies may provide more accurate simulations of pre-whaling little auk abundance by including changes in the interspecific competitive conditions: top-down control caused by fisheries [74] and bottom-up effects of climate on the Arctic food web [75,76]. By exerting demographic pressure on zooplanktivorous fish, modern Arctic fisheries may indeed have disrupted competitive conditions for zooplanktivorous predators and further favoured the demographic rebound of little auks. In contrast, the effects of anthropogenic climate change on secondary production are less well understood. It has been suggested that climate change may already favour small copepod species, thereby diminishing resources for little auks that preferentially target large species [77]. However, we also know that climate change is causing a complex spatial restructuring of Arctic secondary production, leading both to declines and increases in productivity depending on the region, currents and climatic scenarios [78]. Such impacts at the other end of this food chain without giants are yet to be understood.

Finally, and looking back in time, palaeoecological studies may provide further insights of the past changes in little auk abundance, as studies of the soil around breeding colonies (e.g. carbon dating of organic residues, quantification of pollen from plants associated with guano) can be used to trace the historical evolution of little auk colonies over the last 500 years [45,79,80]. Their results can prove useful to testing the predictions of our model.

## Data Availability

Simulations of the global distribution of copepod biomass used as inputs in the model are available at [81]. The General Bathymetric Chart of the Oceans dataset is available at [82]. Data and R script are accessible at [83]. Supplementary material is available online [84].
